# Associations of serum n–3 and n–6 polyunsaturated fatty acids with prevalence and incidence of nonalcoholic fatty liver disease

**DOI:** 10.1093/ajcn/nqac150

**Published:** 2022-06-01

**Authors:** Tiia N K Mäkelä, Tomi-Pekka Tuomainen, Sari Hantunen, Jyrki K Virtanen

**Affiliations:** Institute of Clinical Medicine, University of Eastern Finland, Finland; Institute of Public Health and Clinical Nutrition, University of Eastern Finland, Kuopio, Finland; Institute of Public Health and Clinical Nutrition, University of Eastern Finland, Kuopio, Finland; Institute of Public Health and Clinical Nutrition, University of Eastern Finland, Kuopio, Finland

**Keywords:** nonalcoholic fatty liver disease, n–3, n–6, polyunsaturated fatty acids, liver disease, fatty liver, population study

## Abstract

**Background:**

Nonalcoholic fatty liver disease (NAFLD) is a major cause of liver diseases worldwide, and lifestyle and diet are significant factors in its development. Recent studies have suggested that dietary fat quality is associated with the development of NAFLD.

**Objectives:**

Our purpose was to investigate the cross-sectional and longitudinal associations of serum n–3 (ω-3) and n–6 (ω-6) PUFAs with NAFLD among middle-aged and older men and women from eastern Finland. We also investigated the associations of estimated Δ5-desaturase and Δ6-desaturase activities, enzymes involved in PUFA metabolism, with NAFLD.

**Methods:**

After exclusions, the cross-sectional analyses included 1533 men examined in 1984–1989 and 674 men and 870 women examined in 1998–2001 in the Kuopio Ischaemic Heart Disease Risk Factor Study. The longitudinal analyses included 520 men examined in 1991–1993 and 301 men and 466 women examined in 2005–2008. Fatty liver index (FLI) was used as a surrogate for NAFLD. Hepatic steatosis was defined as FLI >60. ANCOVA and logistic regression were used for analyses.

**Results:**

In the longitudinal analyses, participants with higher serum concentrations of total n–6 PUFA and linoleic acid, the major n–6 PUFA, had markedly lower FLI and lower odds for hepatic steatosis (e.g., odds ratios for incident hepatic steatosis in the highest compared with lowest quartiles were ≤0.41), whereas serum γ-linolenic acid concentration was associated with a higher FLI and higher odds for hepatic steatosis. The associations with the other PUFAs were generally weaker and nonsignificant. In the cross-sectional analyses, also the long-chain n–3 PUFAs had inverse associations. In most analyses, high estimated Δ5-desaturase activity was associated with lower risk and high estimated Δ6-desaturase activity with higher risk for NAFLD.

**Conclusions:**

In middle-aged and older Finnish adults, higher serum concentrations of total n–6 PUFAs and linoleic acid were associated with lower odds for future NAFLD.

## Introduction

Nonalcoholic fatty liver disease (NAFLD) is the predominant cause of chronic liver disease worldwide (1). It is strongly associated with metabolic risk factors obesity, dyslipidemia, and insulin resistance and with lifestyle diseases such as metabolic syndrome, type 2 diabetes, and cardiovascular disease (2, 3). Lifestyle is a significant factor in the progression of metabolic diseases, and the current main treatment for NAFLD is lifestyle modification that aims for weight loss (4).

**FIGURE 1:**
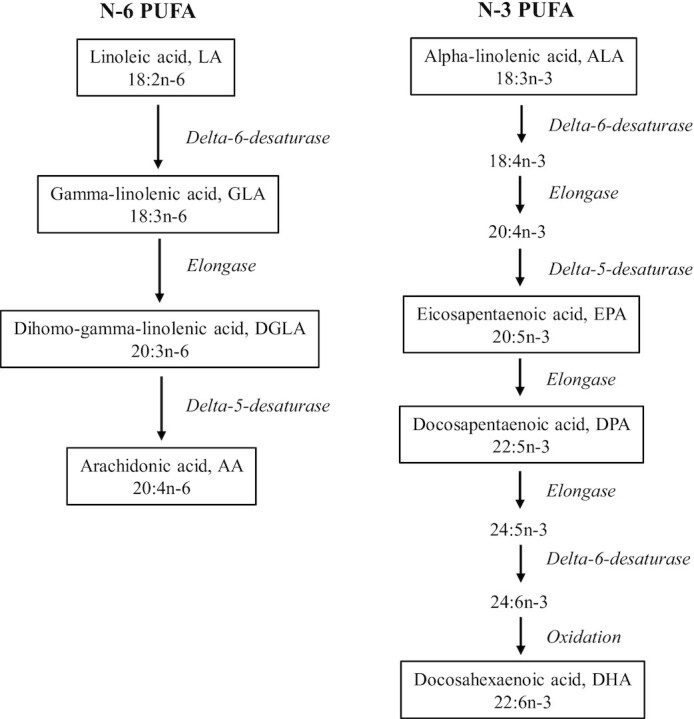
Polyunsaturated fatty acid metabolism.

Dietary intakes of SFAs and PUFAs are shown to affect metabolic diseases, including NAFLD (5, 6). For example, eicosanoids derived from PUFAs can act as ligands for transcription factors, such as peroxisome proliferator–activated receptors (PPARs), which promote fatty acid oxidation and hence decrease fat accumulation into the liver (7, 8). In experimental studies, overfeeding SFA caused lipid accumulation into the liver compared with overfeeding with PUFAs (9–11), although not all studies agree with these results (12). The PUFAs are categorized into n–3 and n–6 PUFAs. In addition to being obtained from diet, these fatty acids, except for the essential fatty acids linoleic acid (C18:2n–6, LA) and α-linolenic acid (C18:3n–3, ALA), are also synthesized endogenously. Important enzymes catalyzing these processes are Δ6-desaturase (D6D) and Δ5-desaturase (D5D) (**Figure 1**).

Experimental studies with long-chain n–3 PUFA supplements (fish oil) have shown beneficial effects on liver fat in patients with NAFLD (13, 14), and increase in LA intake has prevented liver fat accumulation (9–11). In these trials, the n–6 PUFA intake (10–15% of energy from LA) has been higher than what is commonly consumed, so the results may not be directly generalizable to normal healthy populations with typical diets.

The observational evidence of the associations of the n–6 PUFAs with NAFLD is limited and mostly cross-sectional. In 2 studies, serum concentrations of n–6 and n–3 PUFAs were lower in people with steatosis compared with people with normal liver, and the inverse associations with NAFLD were stronger with n–6 PUFAs compared with n–3 PUFAs (15, 16). In a study with an overweight but generally healthy population, serum ALA and especially LA correlated with decreased liver fat (17). Another study found that the other PUFAs associated inversely with NAFLD, but the concentrations of the minor n–6 PUFAs dihomo-γ-linolenic acid (C20:3n–6, DGLA) and γ-linolenic acid (C18:3n–6, GLA) were higher in people with NAFLD or nonalcoholic steatohepatitis (NASH) (16). In the few prospective studies, total PUFA, n–6 PUFA, LA, and ALA associated with decreased risk for NAFLD (18), and the n–3 PUFAs EPA (C20:5n–3) and DHA (C22:6n–3) inversely associated with NAFLD (19).

Our purpose was to add to the limited data available on the extent to which serum n–3 and n–6 PUFA concentrations are associated with the development of NAFLD. We also investigated the associations of estimated D5D and D6D activities with the fatty liver index (FLI). There are only few data from population-based studies regarding how these enzyme activities associate with NAFLD (16, 17, 20).

## Methods

### Study population

The Kuopio Ischaemic Heart Disease Risk Factor Study (KIHD) is a prospective population-based cohort study from eastern Finland, designed primarily to investigate risk factors for cardiovascular disease, atherosclerosis, and related outcomes in a population-based sample of males from eastern Finland (21). Other outcomes, such as NAFLD, can be regarded as secondary outcomes. The KIHD study adhered to the Declaration of Helsinki, and it has an approval of the Research Ethics Committee of the University of Kuopio. All the participants gave a written informed consent for participation.

**FIGURE 2:**
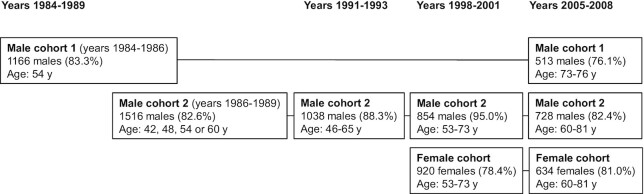
The timeline of the Kuopio Ischaemic Heart Disease Risk Factor Study (KIHD). The figure shows the total numbers of volunteers who participated in each examination round. The percentages in brackets show the proportion of those who participated in the examination rounds, among all eligible participants. For example, for the male cohort 1, 1399 males aged 54 y were eligible to participate in the baseline examinations of the study in 1984–1986. Of the 1399 men, 1166 (83.3%) agreed to participate in the examinations.

The baseline examinations in 1984–1989 were conducted for 2 male cohorts, a total of 2682 males who were from the city of Kuopio and the surrounding rural neighborhoods. Most of the males from the second cohort were examined again in the follow-up visits in 1991–1993 and in 1998–2001. The examinations in 1998–2001 were also the baseline for a female cohort of 920 postmenopausal women from the same area. In 2005–2008, all men, from both baseline male cohorts, and all women were invited for the final KIHD study visit. **Figure 2** shows the total numbers of participants in each examination round.

**FIGURE 3:**
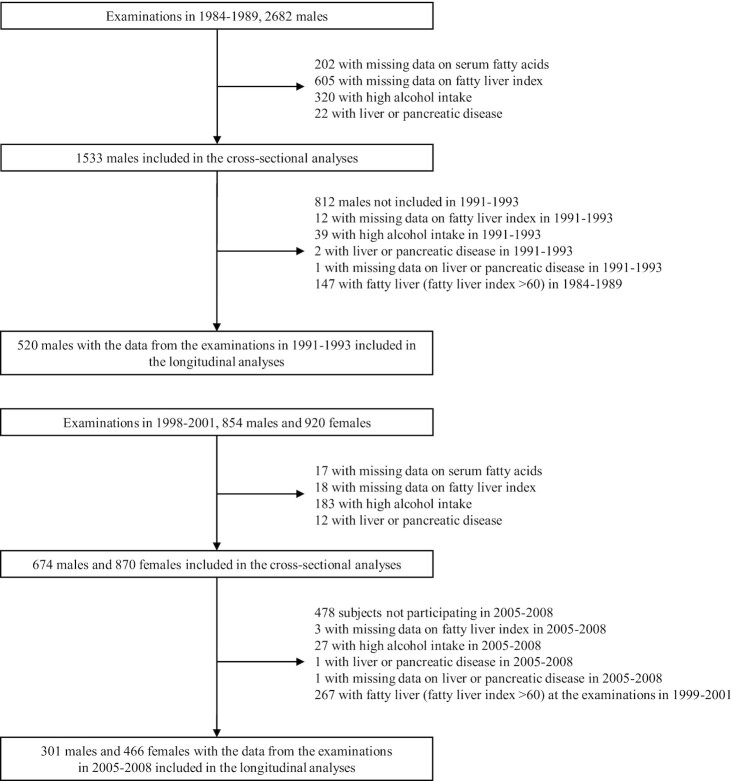
The number of participants included in the analyses.

From the analyses, we excluded participants with high alcohol intake (>20 g/d) or participants with missing data on serum fatty acids or on FLI or who had a diagnosis of a liver disease at any of the examinations. The numbers of participants in the analyses are shown in **Figure 3**.

### Measurements

Venous blood samples were collected between 08:00 and 10:00 at the examinations. Participants were instructed to abstain from ingesting alcohol for 3 d and from smoking and eating for 12 h prior to giving the sample. Detailed determinations of medical history and medications, serum lipids and lipoprotein, smoking, alcohol intake, and blood pressure have been published (22). Hypertension was defined as blood pressure >140/90 mmHg or treatment for hypertension. Diabetes was defined as a self-reported physician-set diagnosis of diabetes and/or fasting plasma glucose ≥7.0 mmol/L or, at the follow-up study visits, 2-h oral glucose tolerance test plasma glucose ≥11.1 mmol/L. Physical activity was evaluated based on the 12-mo leisure-time physical activity questionnaire and expressed as kcal/d (23). The most common leisure-time physical activities were recorded, including the average duration, intensity, and frequency of each activity. Education was assessed in years by using self-administered questionnaire. Dietary intakes were assessed by instructed 4-d food recording (24). Waist circumference, weight, and height were measured at the study visit. BMI was calculated as the ratio of weight in kilograms to height in meters squared (kg/m^2^).

### FLI

**FIGURE 4:**
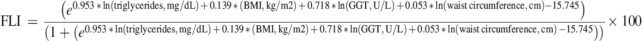
The mathematical formula for the fatty liver index (FLI). GGT, γ-glutamyl-transferase.

For defining NAFLD, we have used FLI, which is a mathematic formula based on BMI, waist circumference, and serum triglyceride and γ-glutamyl-transferase concentrations for predicting the presence of liver fat (**Figure 4**). FLI <30 rules out and FLI ≥60 rules in hepatic steatosis (25, 26).

### Serum fatty acids and desaturases

Serum fatty acids in the samples from 1984–1989 were measured in 1991 from samples that had been stored at –80°C in 1 gas chromatographic run (Hewlett Packard 5890 Series II with flame ionization detector and 7673 autosampler), as described previously (27). Serum fatty acids were extracted with chloroform-methanol. The chloroform phase was evaporated and treated with sodium methoxide, which methylated esterified fatty acids. Quantification was carried out with reference standards purchased from NU-Check Prep Inc. Each analyte had an individual reference standard, and an internal standard was eicosane. Fatty acids were chromatographed in an NB-351 capillary column (HNU-Nordion) by a Hewlett-Packard 5890 Series II gas chromatograph (Hewlett-Packard Company; since 1999 Agilent Technologies) with a flame ionization detector. Results are presented as a proportion of total serum fatty acids. In the 1984–1989 samples, the interassay CV for repeated measurements was 8.7% for LA, 11.6% for GLA, 8.3% for DGLA, 9.9% for arachidonic acid (C20:4n–6, AA), 5.8% for ALA, 5.9% for EPA, 9.2% for docosapentaenoic acid (C22:5n–3, DPA), and 5.7% for DHA. In the 1998–2001 samples, the CV was 5.8% for LA, 4.9% for AA, 10.6% for GLA, 5.5% for DGLA, 5.8% for ALA, 18:3n–3, 5.9% for EPA, 9.2% for DPA, and 5.7% for DHA.

Desaturase enzyme activities were estimated as the ratio of product to precursor (28). Estimated D6D activity was calculated by dividing the concentration of GLA by LA concentration and estimated D5D activity by dividing the AA concentration by DGLA concentration.

### Statistical analysis

Data were analyzed with SPSS version 27 (Statistical Package for the Social Sciences) software (IBM Corp.). The multivariable-adjusted associations with FLI were estimated with ANCOVA. Logistic regression was used to estimate the odds for prevalence and incidence of hepatic steatosis (FLI >60). Quartiles of the serum PUFA concentrations and estimated desaturase enzyme activities were used in the analyses.

The confounders were selected based on established risk factors for NAFLD or on previously published associations with NAFLD. Model 1 was adjusted for age (years) and examination year. In the analyses that included both men and women, sex was also included in model 1. The multivariable model, model 2, was adjusted for model 1 and leisure-time physical activity (kcal/d), smoking (never smoker, previous smoker, current smoker <20 cigarettes/d, and current smoker ≥20 cigarettes/d), and intakes of alcohol (g/wk), energy (kcal/d), carbohydrates [percentage of energy (E%)], and SFAs (E%). All *P* values were 2-tailed (α = 0.05). All quantitative variables were entered as continuous variables in the models. Missing values in covariates were replaced with means of the study population (<2.5% of the values). Linear trends across quartiles were assessed after assigning the median PUFA or desaturase enzyme activity value for the categories and then treating that as a continuous variable in the statistical models. Statistical significance of the potential interactions by sex was assessed by stratified analysis and likelihood ratio tests using a multiplicative interaction term.

## Results

### Baseline characteristics

The mean age of the cohort was 53.1 ± 5.4 y at baseline in 1984–1989 and 63.1 ± 6.4 y at the examinations in 1998–2001. The examinations in 1998–2001 included women, who composed 56% of the cohort. Data on the participants’ lifestyle factors, diseases, dietary intakes, and serum PUFA concentrations are given in **Table 1**. **Supplemental Table 1** shows the baseline characteristics according to the quartiles of the serum total n–3 and n–6 PUFA concentrations at the baseline examinations in 1984–1989 and **Supplemental Table 2** similarly at the examinations in 1998–2001. In general, participants with higher total n–3 and n–6 PUFA concentrations had higher education and leisure-time physical activity. They smoked less and were overall healthier (i.e., had lower prevalence of type 2 diabetes, metabolic syndrome, cardiovascular disease, or hypertension). Alcohol intake was higher among those with higher serum n–3 PUFA concentrations. Differences in dietary intakes were in general small, but higher serum concentrations of both n–3 and n–6 PUFAs were associated with lower saturated fat intake and higher intake of PUFAs.

**TABLE 1:** Baseline characteristics of the Kuopio Ischaemic Heart Disease Risk Factors Study in 1984–1989 and 1998–2001^1^

Characteristic	Men, 1984–1989 (*n* = 1533)	Men and women, 1998–2001 (*n* = 1544)
Age, y	53.1 ± 5.4	63.1 ± 6.4
Sex, male, %	100	44
Education, y	8.7 ± 3.4	9.5 ± 3.4
Leisure-time physical activity, kcal/d	142 ± 177	188 ± 205
Current smoker, %	25.0	10.2
Diabetes, %	5.1	11.4
Metabolic syndrome, %	12.5	29.7
Cardiovascular disease, %	36.3	44.4
Hypertension, %	57.7	64.8
Alcohol intake, g/wk	34.2 ± 36.3	24.8 ± 33.6
FLI components		
BMI, kg/m^2^	26.8 ± 3.4	27.8 ± 4.5
Waist circumference, cm	90.8 ± 9.8	91.7 ± 12.2
Triglycerides, mmol/L	1.3 ± 0.8	1.3 ± 0.7
GGT, U/L	25.6 ± 23.9	28.4 ± 35.6
Dietary intakes		
Energy, kcal/d	2424 ± 622	1815 ± 557
SFAs, E%	18.1 ± 4.2	14.1 ± 3.3
MUFAs, E%	11.8 ± 2.2	10.9 ± 2.4
PUFAs, E%	4.6 ± 1.3	4.9 ± 1.4
*Trans* fatty acids, E%	1.1 ± 0.4	1.0 ± 0.4
Carbohydrates, E%	43.8 ± 6.2	48.0 ± 6.3
Protein, E%	15.9 ± 2.6	17.3 ± 2.9
Serum n–6 polyunsaturated fatty acids		
Linoleic acid (C18:2n–6), %	26.68 ± 4.52	24.42 ± 3.54
γ-Linolenic acid (C18:3n–6), %	0.28 ± 0.11	0.34 ± 0.14
Dihomo-γ-linolenic acid (C20:3n–6), %	1.35 ± 0.32	1.35 ± 0.28
Arachidonic acid (C20:4n–6), %	4.76 ± 1.00	5.79 ± 1.21
Serum n–3 polyunsaturated fatty acids		
α-Linolenic acid (C18:3n–3), %	0.75 ± 0.24	0.97 ± 0.29
EPA (C20:5n–3), %	1.61 ± 0.89	1.59 ± 0.87
Docosapentaenoic acid (C22:5n–3), %	0.56 ± 0.11	0.76 ± 0.16
DHA (C22:6n–3), %	2.47 ± 0.74	2.70 ± 0.90

1 All values are means ± SDs or percentages. E%, percentage of energy; FLI, fatty liver index; GGT, γ-glutamyl-transferase.

### n–3 and n–6 PUFA concentrations, desaturase enzyme activities, and incident FLI

**Table 2** shows the mean values of FLI and odds for hepatic steatosis among men at the examinations in 1991–1993 in quartiles of the serum n–3 and n–6 PUFAs and estimated desaturase enzyme activities measured in 1984–1989 at the baseline examinations. After adjustment for age and examination year (model 1), those in the highest compared with the lowest serum total n–6 PUFA quartile had 28% lower FLI on average (mean difference: 11.8 units; 95% CI: 6.5, 17.2 units; *P*-trend across quartiles < 0.001). The odds for FLI >60 (i.e., hepatic steatosis) was 71% (95% CI: 41%, 86%) lower in the highest compared with the lowest quartile (*P*-trend < 0.001). Further adjustments for potential confounders (model 2) had little impact on the associations (Table 2). When the n–6 PUFAs were investigated individually, those in the highest compared with the lowest quartile of serum LA concentration had 31% lower FLI (mean difference between quartiles: 13.4 units; 95% CI: 8.0, 18.8 units; *P*-trend < 0.001) and 73% lower odds for hepatic steatosis (95% CI: 43%, 87%; *P*-trend < 0.001) (model 1), with little change in the estimates after further adjustments (model 2, Table 2). Other PUFAs were not associated with FLI (Table 2). Higher estimated D5D activity associated with lower FLI and lower odds for hepatic steatosis, whereas no association was found with estimated D6D activity (Table 2).

**TABLE 2:** Mean values of fatty liver index and odds for hepatic steatosis in 1991–1993 in quartiles of serum n–3 and n–6 PUFAs and Δ6-desaturase and Δ5-desaturase activities measured in 1984–1989

Characteristic	Quartile of serum PUFAs^1^				*P*-trend	Odds ratio (95% CI) for hepatic steatosis (FLI >60) in the highest vs. lowest serum PUFA quartile^2^	*P*-trend
	1 (*n* = 130)	2 (*n* = 130)	3 (*n* = 130)	4 (*n* = 130)			
Total n–6 PUFA, %	<31.7	31.7–34.6	34.7–37.4	>37.4			
Model 1	42.7 (39.0, 46.5)	44.2 (40.4, 47.9)	31.8 (28.0, 35.5)	30.9 (27.2, 34.7)	<0.001	0.29 (0.14, 0.59)	<0.001
Model 2	43.1 (39.3, 46.8)	44.2 (40.5, 47.9)	32.0 (28.2, 35.7)	30.4 (26.6, 34.2)	<0.001	0.27 (0.13, 0.57)	<0.001
FLI >60, *n* (%)	34 (26.2)	28 (21.5)	19 (14.6)	12 (9.2)			
LA, %	<25.3	25.3–28.0	28.1–30.7	>30.7			
Model 1	43.9 (40.1, 47.7)	39.9 (36.1, 43.7)	35.4 (31.6, 39.2)	30.5 (26.7, 34.3)	<0.001	0.27 (0.13, 0.57)	<0.001
Model 2	43.7 (39.8, 47.5)	40.4 (36.6, 44.1)	35.4 (31.7, 39.2)	30.1 (26.3, 33.9)	<0.001	0.27 (0.13, 0.58)	<0.001
FLI >60, *n* (%)	33 (25.4)	26 (20.0)	23 (17.7)	11 (8.5)			
GLA, %	<0.19	0.19–0.26	0.27–0.35	>0.35			
Model 1	37.7 (33.8, 41.6)	35.6 (31.7, 39.5)	38.3 (34.4, 42.1)	38.0 (34.1, 41.9)	0.717	0.84 (0.45, 1.59)	0.800
Model 2	37.6 (33.8, 41.5)	35.8 (32.0, 39.7)	37.9 (34.1, 41.8)	38.2 (34.4, 42.1)	0.661	0.88 (0.46, 1.68)	0.840
FLI >60, *n* (%)	26 (20.0)	20 (15.4)	25 (19.2)	22 (16.9)			
DGLA, %	<1.1	1.1–1.3	1.4–1.5	>1.5			
Model 1	38.0 (34.2, 41.9)	35.4 (31.6, 39.3)	36.5 (32.7, 40.4)	39.6 (35.7, 43.5)	0.510	1.16 (0.64, 2.12)	0.524
Model 2	36.6 (32.8, 40.5)	35.0 (31.1, 38.8)	37.8 (33.9, 41.6)	40.2 (36.4, 44.1)	0.131	1.37 (0.74, 2.54)	0.237
FLI >60, *n* (%)	26 (20.0)	18 (13.8)	20 (15.4)	29 (22.3)			
AA, %	<4.1	4.1–4.8	4.9–5.5	>5.5			
Model 1	38.7 (34.8, 42.6)	38.6 (34.7, 42.5)	35.6 (31.8, 39.5)	36.7 (32.8, 40.6)	0.337	0.66 (0.35, 1.23)	0.197
Model 2	39.2 (35.4, 43.0)	39.3 (35.5, 43.2)	36.1 (32.3, 39.9)	35.0 (31.1, 38.9)	0.078	0.55 (0.29, 1.05)	0.069
FLI >60, *n* (%)	30 (23.1)	21 (16.2)	21 (16.2)	21 (16.2)			
Total n–3 PUFA, %	<4.3	4.3–5.0	5.1–6.0	>6.0			
Model 1	35.7 (31.8, 39.6)	35.8 (31.9, 39.7)	40.5 (36.6, 44.3)	37.6 (33.8, 41.5)	0.348	0.90 (0.46, 1.75)	0.923
Model 2	36.5 (32.6, 40.3)	36.2 (32.4, 40.1)	40.2 (36.4, 44.0)	36.7 (32.9, 40.6)	0.769	0.80 (0.40, 1.57)	0.752
FLI >60, *n* (%)	22 (16.9)	18 (13.8)	33 (25.4)	20 (15.4)			
ALA, %	<0.65	0.65–0.77	0.78–0.96	>0.96			
Model 1	38.5 (34.6, 42.4)	36.4 (32.5, 40.3)	38.9 (35.0, 42.8)	35.8 (31.9, 39.7)	0.483	0.83 (0.43, 1.58)	0.700
Model 2	37.4 (33.5, 41.3)	36.5 (32.7, 40.3)	39.1 (35.3, 43.0)	36.6 (32.7, 40.4)	0.942	0.94 (0.48, 1.82)	0.996
FLI >60, *n* (%)	26 (20.0)	21 (16.2)	24 (18.5)	22 (16.9)			
EPA, %	<1.0	1.0–1.3	1.4–1.8	>1.8			
Model 1	35.6 (31.7, 39.5)	37.3 (33.4, 41.2)	36.7 (32.8, 40.6)	39.9 (36.1, 43.8)	0.134	1.33 (0.70, 2.55)	0.521
Model 2	36.4 (32.5, 40.3)	37.4 (33.5, 41.2)	36.7 (32.9, 40.5)	39.1 (35.3, 43.0)	0.344	1.21 (0.62, 2.35)	0.757
FLI >60, *n* (%)	20 (15.4)	25 (19.2)	23 (17.7)	25 (19.2)			
DPA, %	<0.49	0.49–0.55	0.56–0.61	>0.61			
Model 1	40.9 (37.0, 44.8)	37.2 (33.4, 41.1)	35.8 (31.9, 39.7)	35.6 (31.7, 39.5)	0.056	0.56 (0.29, 1.07)	0.080
Model 2	40.4 (36.6, 44.3)	37.4 (33.6, 41.2)	36.2 (32.4, 40.0)	35.6 (31.7, 39.4)	0.076	0.55 (0.28, 1.07)	0.083
FLI >60, *n* (%)	30 (23.1)	23 (17.7)	22 (16.9)	18 (13.8)			
DHA, %	<1.9	1.9–2.3	2.4–2.9	>2.9			
Model 1	35.7 (31.9, 39.6)	36.7 (32.8, 40.5)	40.2 (36.3, 44.0)	37.0 (33.1, 40.9)	0.537	0.88 (0.45, 1.74)	0.761
Model 2	37.3 (33.4, 41.3)	36.2 (32.4, 40.1)	40.2 (36.4, 44.1)	35.8 (31.9, 39.7)	0.795	0.68 (0.33, 1.40)	0.344
FLI >60, *n* (%)	21 (16.2)	24 (18.5)	29 (22.3)	19 (14.6)			
D6D activity	<0.007	0.007–0.009	0.010–0.013	>0.013			
Model 1	35.7 (31.8, 39.6)	35.7 (31.8, 39.6)	37.9 (34.0, 41.8)	40.3 (36.4, 44.2)	0.065	1.38 (0.74, 2.56)	0.246
Model 2	36.0 (32.1, 39.8)	35.5 (31.7, 39.4)	37.6 (33.8, 41.5)	40.5 (36.6, 44.3)	0.063	1.39 (0.74, 2.62)	0.227
FLI >60, *n* (%)	22 (16.9)	21 (16.2)	22 (16.9)	28 (21.5)			
D5D activity	<3.1	3.1–3.5	3.6–4.4	>4.4			
Model 1	39.9 (36.0, 43.7)	38.6 (34.8, 42.5)	36.9 (33.0, 40.7)	34.2 (30.4, 38.1)	0.033	0.59 (0.32, 1.10)	0.083
Model 2	41.7 (37.9, 45.5)	39.6 (35.8, 43.4)	36.8 (33.0, 40.6)	31.5 (27.6, 35.4)	<0.001	0.40 (0.20, 0.78)	0.006
FLI >60, *n* (%)	32 (24.6)	24 (18.5)	16 (12.3)	21 (16.2)			

1 Values are means (95% CIs) from ANCOVA unless otherwise indicated. AA, arachidonic acid (C20:4n–6); ALA, α-linolenic acid (C18:3n–3); D5D, Δ5-desaturase; D6D, Δ6-desaturase; DGLA, dihomo-γ-linolenic acid (C20:3n–6); DHA, docosahexaenoic acid (C22:6n–3); E%, percentage of energy; FLI, fatty liver index; GLA, γ-linolenic acid (C18:3n–6); LA, linoleic acid (C18:2n–6).

2 Values are odds ratios (95% CIs) from the logistic regression. Model 1 adjusted for age and examination year. Model 2 adjusted for model 1 and leisure-time physical activity (kcal/d), smoking (never smoker, previous smoker, current smoker <20 cigarettes/d, and current smoker ≥20 cigarettes/d), and intakes of alcohol (g/wk), energy (kcal/d), carbohydrates (E%), and SFAs (E%).

**Table 3** shows the associations of the n–3 and n–6 PUFA concentrations and desaturase enzyme activities measured in 1998–2001 with FLI and odds for hepatic steatosis among men and women in 2005–2008. There was no evidence that the associations would be appreciably different between men and women (*P*-interaction > 0.05). The total n–6 PUFA and LA concentrations again had strong inverse associations with FLI and odds for hepatic steatosis. In these longitudinal analyses, the serum GLA and DGLA concentrations associated with higher FLI and higher odds for hepatic steatosis (Table 3). For example, those in the highest compared with the lowest serum GLA quartile had 32% higher FLI (mean difference between quartiles: 8.7 units; 95% CI: 4.4, 12.9 units; *P*-trend < 0.001) after adjustment for age, sex, and examination year (model 1). The odds for hepatic steatosis was 123% (95% CI: 15%, 394%) higher in the highest compared with the lowest quartile (*P*-trend = 0.04). The results remained relatively similar after further adjustments (model 2, Table 3). Among the n–3 PUFAs, only serum DHA concentration had an inverse association with FLI. No association was found with serum AA, ALA, EPA, or DPA concentrations. Higher estimated D6D activity had a strong association with higher FLI and higher odds for hepatic steatosis (*P-*trend < 0.05), whereas higher estimated D5D activity had a strong association with lower FLI (*P-*trend < 0.004).

**TABLE 3:** Mean values of fatty liver index and odds for hepatic steatosis in examinations in 2005–2008 in quartiles of serum n–3 and n–6 PUFAs and Δ6-desaturase and Δ5-desaturase activities measured in 1998–2001

Characteristic	Quartile of serum PUFAs^1^				*P*-trend	Odds ratio (95% CI) for hepatic steatosis (FLI >60) in the highest vs. lowest serum PUFA quartile^2^	*P*-trend
	1 (*n* = 191)	2 (*n* = 192)	3 (*n* = 192)	4 (*n* = 192)			
Total n–6 PUFA, %	<31.4	31.4–33.2	33.3–35.0	>35.0			
Model 1	40.1 (37.1, 43.1)	33.0 (30.0, 36.0)	31.8 (28.8, 34.8)	26.3 (23.3, 29.3)	<0.001	0.30 (0–15, 0.59)	<0.001
Model 2	40.1 (37.1, 43.1)	33.0 (30.0, 35.9)	32.1 (29.1, 35.0)	26.0 (23.0, 29.0)	<0.001	0.29 (0.15, 0.57)	<0.001
FLI >60, *n* (%)	37 (19.4)	27 (14.1)	23 (12.0)	13 (6.8)			
LA, %	<23.5	23.5–25.5	25.6–27.6	>27.6			
Model 1	40.1 (37.1, 43.1)	34.2 (31.2, 37.1)	31.5 (28.5, 34.5)	25.4 (22.4, 28.4)	<0.001	0.37 (0.19, 0.71)	0.002
Model 2	39.8 (36.8, 42.7)	34.3 (31.4, 37.3)	32.0 (29.0, 34.9)	25.1 (22.1, 28.1)	<0.001	0.37 (0.19, 0.73)	0.003
FLI >60, *n* (%)	35 (18.3)	28 (14.6)	22 (11.5)	15 (7.8)			
GLA, %	<0.23	0.23–0.31	0.32–0.41	>0.41			
Model 1	26.8 (23.8, 29.8)	35.0 (31.9, 38.0)	33.9 (30.8, 36.9)	35.5 (32.4, 38.5)	<0.001	2.23 (1.15, 4.34)	0.040
Model 2	26.1 (23.1, 29.1)	35.0 (32.0, 37.9)	34.2 (31.2, 37.2)	35.8 (32.8, 38.8)	<0.001	2.51 (1.27, 4.93)	0.020
FLI >60, *n* (%)	15 (7.9)	28 (14.6)	28 (14.6)	29 (15.1)			
DGLA, %	<1.2	1.2–1.3	1.4–1.5	>1.5			
Model 1	26.8 (23.7, 29.8)	33.8 (30.8, 36.8)	34.3 (31.3, 37.3)	36.2 (33.2, 39.2)	<0.001	1.93 (1.00, 3.72)	0.128
Model 2	25.6 (22.6, 28.7)	33.8 (30.8, 36.8)	34.6 (31.6, 37.5)	37.1 (34.1, 40.1)	<0.001	2.31 (1.17, 4.56)	0.044
FLI >60, *n* (%)	16 (8.4)	31 (16.1)	25 (13.0)	28 (14.6)			
AA, %	<5.1	5.1–5.8	5.9–6.7	>6.7			
Model 1	32.5 (29.4, 35.5)	31.1 (28.1, 34.2)	33.2 (30.1, 36.3)	34.3 (31.2, 37.4)	0.273	1.19 (0.66, 2.14)	0.412
Model 2	33.1 (30.1, 36.2)	31.3 (28.2, 34.3)	33.2 (30.1, 36.2)	33.5 (30.5, 36.6)	0.643	1.05 (0.57, 1.91)	0.697
FLI >60, *n* (%)	25 (13.1)	20 (10.4)	25 (13.0)	30 (15.6)			
Total n–3 PUFA, %	<4.9	4.9–5.8	5.9–7.0	>7.0			
Model 1	32.8 (29.7, 35.8)	33.1 (30.0, 36.1)	34.7 (31.7, 37.8)	30.5 (27.5, 33.6)	0.324	1.12 (0.60, 2.12)	0.773
Model 2	34.2 (31.0, 37.3)	33.4 (30.4, 36.5)	33.9 (30.9, 37.0)	29.6 (26.5, 32.7)	0.041	1.01 (0.52, 1.95)	0.900
FLI >60, *n* (%)	21 (11.0)	26 (13.5)	30 (15.6)	23 (12.0)			
ALA, %	<0.79	0.79–0.94	0.95–1.12	>1.12			
Model 1	35.3 (32.2, 38.4)	31.3 (28.2, 34.3)	33.1 (30.1, 36.2)	31.5 (28.4, 34.5)	0.152	0.61 (0.34, 1.10)	0.120
Model 2	35.5 (32.4, 38.5)	31.3 (28.2 34.3)	33.2 (30.1, 36.2)	31.2 (28.2, 34.3)	0.112	0.60 (0.32, 1.09)	0.113
FLI >60, *n* (%)	33 (17.3)	22 (11.5)	23 (12.0)	22 (11.5)			
EPA, %	<1.1	1.1–1.3	1.4–1.9	>1.9			
Model 1	31.5 (28.4, 34.6)	33.6 (30.5, 36.6)	33.0 (30.0, 36.1)	33.0 (30.0, 36.1)	0.638	1.61 (0.85, 3.07)	0.255
Model 2	32.8 (29.7, 35.9)	33.6 (30.5, 36.6)	32.5 (29.5, 35.6)	32.2 (29.1, 35.3)	0.672	1.43 (0.73, 2.78)	0.486
FLI >60, *n* (%)	18 (9.4)	27 (14.1)	29 (15.1)	26 (13.5)			
DPA, %	<0.67	0.67–0.77	0.78–0.87	>0.87			
Model 1	33.4 (30.3, 36.6)	33.4 (30.3, 36.4)	34.5 (31.5, 37.6)	29.8 (26.6, 32.9)	0.151	0.71 (0.37, 1.35)	0.321
Model 2	34.0 (30.9, 37.1)	33.4 (30.4, 36.4)	34.0 (31.0, 37.1)	29.7 (26.6, 32.8)	0.075	0.68 (0.35, 1.32)	0.276
FLI >60, *n* (%)	25 (13.1)	26 (13.5)	28 (14.6)	21 (10.9)			
DHA, %	<2.1	2.1–2.6	2.7–3.3	>3.3			
Model 1	33.9 (30.8, 37.0)	33.9 (30.8, 36.9)	32.2 (29.1, 35.2)	31.1 (28.0, 34.2)	0.146	0.92 (0.50, 1.68)	0.658
Model 2	35.2 (32.1, 38.3)	34.3 (31.3, 37.4)	31.6 (28.6, 34.6)	29.9 (26.9, 33.0)	0.012	0.80 (0.42, 1.51)	0.374
FLI >60, *n* (%)	26 (13.6)	27 (14.1)	23 (12.0)	24 (12.5)			
D6D activity	<0.009	0.009–0.012	0.013–0.017	>0.017			
Model 1	26.2 (23.2, 29.2)	33.9 (30.9, 37.0)	34.8 (31.8, 37.8)	36.2 (33.2, 39.2)	<0.001	2.42 (1.23, 4.77)	0.029
Model 2	25.4 (22.4, 28.4)	34.2 (31.2, 37.2)	35.1 (32.1, 38.1)	36.4 (33.4, 39.4)	<0.001	2.73 (1.36, 5.46)	0.017
FLI >60, *n* (%)	14 (7.3)	28 (14.7)	29 (15.1)	29 (15.1)			
D5D activity	<3.7	3.7–4.4	4.5–5.3	>5.3			
Model 1	37.0 (33.9, 40.0)	32.5 (29.5, 35.5)	31.1 (28.0, 34.1)	30.6 (27.5, 33.6)	0.004	0.76 (0.40, 1.43)	0.472
Model 2	38.1 (35.0, 41.1)	33.0 (30.0, 36.0)	31.0 (28.0, 34.0)	29.1 (26.0, 32.1)	<0.001	0.61 (0.31, 1.17)	0.157
FLI >60, *n* (%)	24 (12.6)	25 (13.0)	31 (16.1)	20 (10.4)			

1 Values are means (95% CIs) from ANCOVA unless otherwise indicated. AA, arachidonic acid (C20:4n–6); ALA, α-linolenic acid (C18:3n–3); D5D, Δ5-desaturase; D6D, Δ6-desaturase; DGLA, dihomo-γ-linolenic acid (C20:3n–6); DHA, docosahexaenoic acid (C22:6n–3); E%, percentage of energy; FLI, fatty liver index; GLA, γ-linolenic acid (C18:3n–6); LA, linoleic acid (C18:2n–6).

2 Values are odds ratios (95% CIs) from the logistic regression. Model 1 adjusted for age, sex, and examination year. Model 2 adjusted for model 1 and leisure-time physical activity (kcal/d), smoking (never smoker, previous smoker, current smoker <20 cigarettes/d, and current smoker ≥20 cigarettes/d), and intakes of alcohol (g/wk), energy (kcal/d), carbohydrates (E%), and SFAs (E%).

### n–3 and n–6 PUFA concentrations, desaturase enzyme activities, and prevalent FLI

Cross-sectional results among the male participants at baseline in 1984–1989 are presented in **Supplemental Table 3**. In the highest compared with the lowest serum total n–6 PUFA quartile, higher serum concentration associated with lower FLI, and the odds for hepatic steatosis was 92% (95% CI: 87%, 95%) lower in the highest compared with the lowest quartile (*P*-trend < 0.001) (model 1). When the n–6 PUFAs were investigated individually, higher LA and AA concentrations were associated with a lower FLI and lower odds for hepatic steatosis, whereas higher GLA and DGLA concentrations were associated with higher FLI. Among the n–3 PUFAs, the total n–3 PUFA, EPA, and DHA had inverse but only modest associations and ALA was not associated with FLI (Supplemental Table 3). In contrast, the inverse association with DPA was stronger. The odds for hepatic steatosis was 66% (95% CI: 52%, 76%) lower in the highest compared with the lowest DPA quartile (*P*-trend < 0.001).

In the cross-sectional analyses in 1998–2001 that included both men and women, the results were similar but even stronger than in the cross-sectional analyses with the data from 1984–1989. The results are presented in **Supplemental Table 4**. Total n–6 and n–3 PUFAs, LA, AA, EPA, DPA, DHA, and estimated D5D activity were strongly associated with lower FLI and lower odds for hepatic steatosis, whereas GLA, DGLA, and estimated D6D activity were associated with higher FLI and higher odds for hepatic steatosis. The associations with the serum concentrations of total n–3 PUFAs (*P*-interaction = 0.01 for FLI and *P*-interaction = 0.03 for odds for hepatic steatosis) and DHA (*P*-interaction = 0.002 for FLI and *P*-interaction = 0.01 for odds for hepatic steatosis) were stronger in women than in men. All other *P* values for interactions were >0.05.

### Additional analyses

Adjusting model 2 for history of diseases that may associate with NAFLD and whose risk may be influenced by n–3 and n–6 PUFAs, including type 2 diabetes, metabolic syndrome, hypertension, and cardiovascular disease, had little impact on the associations (data not shown).

## Discussion

In this population-based study among middle-aged and older men and women, especially higher serum concentrations of total n–6 PUFA and LA were associated with a lower risk of NAFLD. In contrast, serum GLA and DGLA concentrations were associated with higher risk of NAFLD. The inverse associations with the n–3 PUFAs were observed mainly in the cross-sectional analyses, but ALA had no associations. Finally, higher estimated D5D activity was associated with lower risk and higher estimated D6D activity with higher risk of NAFLD.

When comparing macronutrient intakes, a hypercaloric diet rich in n–6 PUFAs has not affected liver fat, whereas overfeeding SFAs has markedly increased liver fat in a healthy population (10). With obese patients, an isocaloric diet high in n–6 PUFAs even reduced liver fat compared with SFA-rich diet (9). In contrast, in a recent study, there was no difference in liver fat accumulation in healthy individuals with eucaloric diets that differed in SFA and PUFA intake (12). n–3 PUFA supplementation has been investigated as a potential treatment for NAFLD, and in randomized controlled trials, supplementation has reduced liver fat in patients with NAFLD (29–31). However, it seems that n–3 PUFA supplements do not cause significant histologic changes when investigating NASH instead of NAFLD (32, 33). The results with patients with NAFLD may not be generalizable to healthy populations. Our results showed that PUFAs, except for the minor n–6 PUFAs GLA and DGLA, were in general inversely associated with fatty liver, n–6 PUFAs more than n–3 PUFAs. This is in line with previous population-based studies (15, 16). In a cross-sectional study with an elderly but generally healthy population, serum LA concentration was inversely associated with liver fat (17). In our study, the strongest associations were observed with total n–6 PUFAs and LA, with both inversely associated with NAFLD. There are limited data from other prospective studies. Another Finnish population-based study among healthy participants showed that serum total n–3 and n–6 PUFAs and LA were associated with lower future risk for developing fatty liver (15). On the other hand, a recent study with the same data showed strong inverse prospective associations between serum total PUFAs, total n–6 PUFAs, LA, and ALA and NAFLD (18). Interestingly, in most of our analyses, GLA and DGLA were associated with higher FLI. This is supported by similar results from other cross-sectional studies (16, 34), together with a prospective study in which a positive trend was observed with GLA and DGLA and fatty liver (18). In a population-based study focusing on n–3 PUFAs, erythrocyte membrane total n–3 PUFAs, EPA, and DHA were inversely associated with NAFLD cross-sectionally, and DHA was associated with NAFLD improvement in the prospective analysis (19).

Some potential mechanisms could explain the observed findings. PUFAs and the eicosanoids derived from them can act as ligands for transcription factors and induce changes in gene expression (8). Fat accumulation into liver is caused by the imbalance between acquisition and disposal of intrahepatic lipids, and gene regulation is assumed to affect hepatic fatty acid metabolism (2, 8). The sterol regulatory element binding protein 1c (SREBP1c) is a transcription factor required for intrahepatic lipid synthesis (de novo lipogenesis) and the PPAR*α* upregulates fatty acid oxidation (7, 35). PUFAs and their eicosanoids suppress the activity of SREBP1c and activate PPAR*α* (7, 8). As a result, liver fat accumulation is decreased, because de novo lipogenesis is diminished and more fatty acids go into oxidation and secretion. SREBP1c and PPAR*γ* expression levels associated with NASH in a population-based study (36). In addition, the eicosanoids derived from PUFA have anti- and proinflammatory effects (8, 37). Overproduction of proinflammatory metabolites has various inflammatory effects, resulting in low-grade inflammation and disease progression, including NAFLD (8). Oxidative stress causes cellular damage and impaired liver function, promotes lipid accumulation, and, with inflammatory signaling, seems to affect the progression from simple steatosis to NASH (35, 38, 39). n–6 PUFA-derived eicosanoids were typically considered proinflammatory and n–3 PUFA eicosanoids anti-inflammatory, but recent research indicates that n–6 PUFA-derived eicosanoids have also anti-inflammatory properties (40), and in experimental studies, even high doses of LA or AA have not increased inflammation (41–43). However, in our study and in other previous studies, LA but not AA has been inversely associated with NAFLD, and in our study, LA had much stronger inverse associations than the n–3 PUFAs. Whether this relates to the fact that LA is the most abundant PUFA in the diet and in the body or whether there are some specific mechanisms other than the anti-inflammatory effects by which LA exerts its actions against liver fat accumulation remains to be elucidated.

In our study, higher estimated D5D activity was associated with lower risk for NAFLD, whereas associations with estimated D6D activity were rather the opposite. The results are in line with most studies (16, 17, 20, 44), and similar associations were also found in the previous KIHD studies with type 2 diabetes and metabolic syndrome (45, 46). D5D and D6D are the limiting enzymes in the PUFA formation (Figure 1). Because these enzymes control the longer-chain PUFA formation, they also regulate the concentrations of eicosanoids derived from them. Higher concentrations of GLA and DGLA and higher estimated D6D activity were associated with higher risk for NAFLD in our study and higher estimated D5D activity with lower risk. These findings could suggest that the associations might be explained by accumulation of GLA and DGLA. However, both GLA and DGLA have been shown to rather have anti-inflammatory properties (40), so production of proinflammatory eicosanoids is not a likely mechanism for the observed associations. There is previous evidence that in obese patients with NAFLD, the activities of both D6D and D5D are reduced, which is related to oxidative stress caused by hepatic steatosis (47). However, in our longitudinal analyses, the participants had a normal liver function at baseline, so NAFLD could not have affected the desaturase activities. More research is needed to elucidate the roles and mechanisms of the desaturase enzymes in the development of NAFLD.

Major strengths of our study include the population-based cohort with both male and female sexes, data from several time points that enabled the longitudinal analyses, extensive examinations of potential confounding factors, and the use of serum fatty acids as the exposure. Use of objective biomarkers eliminates the random error that is often inherited in the subjective methods that are used to assess dietary intakes and that can attenuate the true associations. Using the biomarkers also enabled to investigate the associations of the mainly endogenously produced PUFA, GLA, DGLA, and DPA, as well as the associations with the desaturase enzymes.

Limitations of our study include the use of the FLI algorithm as a proxy for liver fat content and not the imaging methods or liver biopsy specimens, which are considered the gold standard for assessing liver fat (48). However, to estimate the presence of steatosis, FLI has a good diagnostic performance and is described as a good screening tool for NAFLD (49). Another potential weakness is the use of estimated desaturase activities from the product-to-precursor ratio. Although the ratios are used to estimate desaturase activities (28), without directly confirming with measured activities, the ratios may not accurately reflect the hepatic enzyme activities. The number of different analyses was large, which increases the possibility of type II error. However, many of the associations were observed in both cross-sectional and longitudinal analyses, suggesting that the associations did not occur due to chance. Finally, our study included only middle-aged and older Caucasian men and women, so the findings may not be generalizable to younger populations or other ethnicities.

In conclusion, our results suggest that especially the major n–6 PUFA LA may protect against development of NAFLD. This finding is in line with the observed cardiometabolic benefits of LA (50) and indicates that dietary sources of LA, such as nuts, seeds, and many vegetable oils, may be beneficial also in the prevention of NAFLD. The role of the other n–6 and n–3 PUFAs is less clear.

## Supplementary Material

nqac150_Supplemental_File

## Notes

The authors reported no funding received for this study.

Data described in this manuscript will not be made available, because it contains sensitive personal data of the subjects, which cannot be completely anonymized.

Supplemental Tables 1–4 are available from the “Supplementary data” link in the online posting of the article and from the same link in the online table of contents at https://academic.oup.com/ajcn/.

Abbreviations used: AA, arachidonic acid; ALA, α-linolenic acid; D5D, Δ5-desaturase; D6D, Δ6-desaturase; DGLA, dihomo-γ-linolenic acid; DPA, docosapentaenoic acid; E%, percentage of energy; FLI, fatty liver index; GLA, γ-linolenic acid; KIHD, Kuopio Ischaemic Heart Disease Risk Factor Study; LA, linoleic acid; NAFLD, nonalcoholic fatty liver disease; NASH, nonalcoholic steatohepatitis; PPAR, peroxisome proliferator–activated receptor; SREBP1c, sterol regulatory element binding protein 1c.
