# Supplementary material for: Associations of serum n–3 and n–6 polyunsaturated fatty acids with prevalence and incidence of nonalcoholic fatty liver disease
Source: Am J Clin Nutr. 2022 Jun 1;116(3):759–70. doi: 10.1093/ajcn/nqac150 (PMC9437980; doi:10.1093/ajcn/nqac150)
Supplement: nqac150_Supplemental_File [file nqac150_supplemental_file.pdf]

Associations of serum n-3 and n-6 polyunsaturated fatty acids with prevalence and incidence of non-alcoholic fatty liver disease

Mäkelä, et al.

Online Supplemental Material

Supplemental Table 1. Baseline characteristics according to quartiles of serum total n-3 and total n-6 polyunsaturated fatty acids

	Total n-3 PUFA (%)		*P* for trend	Total n-6 PUFA (%)		*P* for trend
	Q1 (<4.3)	Q4 (>6.0)		Q1 (<29.9)	Q4 (>36.2)	
Number of subjects	383	383		383	383	
Age (years)	53.1 ± 5.4	53.0 ± 5.4	0.837	54.0 ± 4.8	52.5 ± 6.0	<0.001
Education (years)	8.5 ± 3.0	9.3 ± 3.7	<0.001	8.4 ± 3.1	9.3 ± 3.7	<0.001
Leisure-time physical activity (kcal/d)	135 ± 173	170 ± 214	0.002	130 ± 164	162 ± 193	0.012
Current smoker (%)	26.4	19.8	0.013	31.6	20.4	0.001
Diabetes (%)	8.9	4.4	0.015	9.9	2.9	<0.001
Metabolic syndrome (%)	16.6	10.7	0.030	34.0	2.6	<0.001
Cardiovascular disease (%)	42.3	34.5	0.131	43.1	33.7	0.009
Hypertension (%)	62.7	57.7	0.221	69.2	49.6	<0.001
Alcohol intake (g/wk)	28.1 ± 34.8	42.2 ± 38.4	<0.001	36.9 ± 38.8	32.4 ± 35.7	0.064
FLI components						
BMI (kg/m^2)	26.9 ± 3.5	27.1 ± 3.3	0.132	28.3 ± 3.9	25.7 ± 2.9	<0.001
Waist circumference (cm)	91.2 ± 10.2	91.3 ± 9.9	0.360	95.4 ± 10.8	87.2 ± 8.2	<0.001
Triglycerides (mmol/L)	1.6 ± 1.0	1.2 ± 0.7	<0.001	2.0 ± 1.1	0.9 ± 0.4	<0.001
GGT (U/L)	26.3 ± 28.3	26.2 ± 24.5	0.781	32.8 ± 32.2	22.5 ± 23.1	<0.001
Dietary intakes						
Energy (kcal/d)	2435 ± 695	2347 ± 582	0.004	2355 ± 641	2473 ± 602	0.003
Saturated fatty acids (E%)	18.4 ± 4.6	17.2 ± 3.8	<0.001	18.9 ± 4.7	17.1 ± 3.6	<0.001
Monounsaturated fatty acids (E%)	12.0 ± 2.3	11.6 ± 2.1	0.040	11.3 ± 2.1	12.4 ± 2.3	<0.001
Polyunsaturated fatty acids (E%)	4.5 ± 1.4	4.8 ± 1.3	0.004	4.1 ± 1.3	5.2 ± 1.3	<0.001
Trans fatty acids (E%)	1.2 ± 0.5	1.0 ± 0.3	<0.001	1.0 ± 0.3	1.2 ± 0.4	<0.001
Carbohydrates (E%)	44.4 ± 6.5	43.4 ± 6.2	0.007	43.1 ± 6.6	44.4 ± 6.0	0.002
Protein (E%)	15.3 ± 2.4	16.9 ± 2.9	<0.001	16.3 ± 3.0	15.8 ± 2.4	0.003

All values are means (SD) or percentages.

Abbreviations: E%, percent of energy; FLI, fatty liver index; GGT, gamma-glutamyl-transferase; PUFA, polyunsaturated fatty acids

Supplemental Table 2. Baseline characteristics according to quartiles of serum total n-3 and total n-6 polyunsaturated fatty acids in the 11-year examinations

	Total n-3 PUFA (%)		*P* for trend	Total n-6 PUFA (%)		*P* for trend
	Q1 (<4.80)	Q4 (>6.83)		Q1 (<29.5)	Q4 (>34.4)	
Number of subjects	386	386		386	386	
Age (years)	62.6 ± 6.5	63.5 ± 6.4	0.194	64.0 ± 6.5	62.2 ± 6.5	<0.001
Gender, male (%)	51	38	0.001	47	47	0.913
Education (years)	9.2 ± 3.3	10.0 ± 3.6	<0.001	9.5 ± 3.6	9.5 ± 3.5	0.870
Leisure-time physical activity (kcal/d)	167 ± 184	207 ± 194	0.013	169 ± 194	216 ± 262	0.001
Current smoker (%)	18.7	5.7	<0.001	11.4	7.8	0.203
Diabetes (%)	11.9	10.4	0.417	20.5	5.4	<0.001
Metabolic syndrome (%)	35.6	19.3	<0.001	61.0	7.3	<0.001
Cardiovascular disease (%)	47.7	38.1	0.003	49.7	43.3	0.019
Hypertension (%)	64.8	61.4	0.265	75.4	56.0	<0.001
Alcohol intake (g/wk)	23.1 ± 32.9	26.2 ± 32.5	0.036	26.3 ± 36.1	21.3 ± 30.0	0.027
FLI components						
BMI (kg/m^2)	27.8 ± 4.6	27.3 ± 4.3	0.047	29.8 ± 4.4	25.6 ± 3.7	<0.001
Waist circumference (cm)	93.0 ± 11.5	89.5 ± 12.5	<0.001	98.0 ± 11.5	86.5 ± 10.8	<0.001
Triglycerides (mmol/L)	1.5 ± 0.9	1.1 ± 0.5	<0.001	2.0 ± 0.9	0.8 ± 0.3	<0.001
GGT (U/L)	25.8 ± 18.3	23.3 ± 19.2	0.169	33.0 ± 41.7	20.8 ± 14.7	<0.001
Dietary intakes						
Energy (kcal/d)	1876 ± 590	1762 ± 499	0.008	1806 ± 555	1888 ± 594	0.017
Saturated fatty acids (E%)	15.0 ± 3.5	13.3 ± 3.1	<0.001	14.1 ± 3.6	14.0 ± 3.0	0.715
Monounsaturated fatty acids (E%)	10.9 ± 2.6	10.8 ± 2.3	0.290	10.5 ± 2.6	11.3 ± 2.5	<0.001
Polyunsaturated fatty acids (E%)	4.6 ± 1.4	5.2 ± 1.4	<0.001	4.7 ± 1.4	5.2 ± 1.5	<0.001
Trans fatty acids (E%)	1.0 ± 0.4	0.9 ± 0.4	0.022	0.9 ± 0.4	1.0 ± 0.4	<0.001
Carbohydrates (E%)	48.3 ± 6.6	47.8 ± 6.1	0.117	48.2 ± 6.5	48.0 ± 6.1	0.891
Protein (E%)	16.4 ± 2.6	18.1 ± 2.8	<0.001	17.6 ± 3.1	17.0 ± 2.8	0.002

All values are means (SD) or percentages

Abbreviations: E%, percent of energy; FLI, fatty liver index; GGT, gamma-glutamyl-transferase; PUFA, polyunsaturated fatty acids

Supplemental table 3. Mean values of fatty liver index and odds for hepatic steatosis in 1984-1989 in quartiles of serum n-3 and n-6 polyunsaturated fatty acids and delta-6-desaturase and delta-5-desaturase activities

	Quartile of serum polyunsaturated fatty acids					Odds ratio (95% CI) for hepatic steatosis (FLI >60) in the highest vs. lowest serum PUFA quartile	
	1 (n=383)	2 (n=383)	3 (n=384)	4 (n=383)	*P* for trend		*P* for trend
Total n-6 PUFA (%)	<29.9	29.9-33.2	33.3-36.2	>36.2			
Model 1	56.4 (54.1, 58.6)[1]	39.7 (37.5, 41.9)	30.1 (27.9, 32.4)	24.9 (22.7, 27.1)	<0.001	0.08 (0.05, 0.13)[2]	<0.001
Model 2	56.9 (54.8, 59.1)	40.2 (38.1, 42.3)	30.3 (28.2, 32.4)	23.7 (21.6, 25.9)	<0.001	0.06 (0.04, 0.10)	<0.001
FLI >60 [n (%)]	185 (48.3)	82 (21.4)	43 (11.2)	29 (7.6)			
LA (%)	<23.7	23.7-26.6	26.7-29.6	>29.6			
Model 1	55.1 (52.8, 57.3)	39.1 (36.9, 41.4)	31.0 (28.7, 33.2)	26.0 (23.7, 28.2)	<0.001	0.10 (0.07, 0.15)	<0.001
Model 2	55.2 (53.0, 57.4)	39.9 (37.7, 42.1)	30.9 (28.7, 33.0)	25.1 (22.9, 27.4)	<0.001	0.08 (0.05, 0.13)	<0.001
FLI >60 [n (%)]	180 (47.0)	79 (20.6)	47 (12.2)	33 (8.6)			
GLA (%)	<0.21	0.21-0.27	0.28-0.35	>0.35			
Model 1	34.7 (32.2, 37.2)	36.7 (34.2, 39.2)	39.9 (37.4, 42.4)	39.8 (37.3, 42.3)	0.001	1.27 (0.90, 1.80)	0.169
Model 2	34.3 (31.8, 36.7)	36.2 (33.8, 38.7)	39.7 (37.3, 42.1)	40.9 (38.5, 43.4)	<0.001	1.46 (1.02, 2.09)	0.032
FLI >60 [n (%)]	74 (19.3)	84 (21.9)	92 (24.0)	89 (23.2)			
DGLA (%)	<1.15	1.15-1.33	1.34-1.52	>1.52			
Model 1	34.5 (32.0, 37.0)	36.0 (33.5, 38.5)	40.0 (37.5, 42.5)	40.7 (38.2, 43.2)	<0.001	1.36 (0.96, 1.92)	0.066
Model 2	32.1 (29.6, 34.5)	35.8 (33.4, 38.2)	40.8 (38.3, 43.2)	42.4 (40.0, 44.9)	<0.001	1.85 (1.28, 2.67)	0.001
FLI >60 [n (%)]	73 (19.1)	82 (21.4)	92 (24.0)	92 (24.0)			
AA (%)	<4.1	4.1-4.7	4.8-5.4	>5.4			
Model 1	46.7 (44.3, 49.2)	39.4 (37.0, 41.9)	33.3 (30.9, 35.8)	31.6 (29.2, 34.1)	<0.001	0.35 (0.25, 0.50)	<0.001
Model 2	47.8 (45.4, 50.2)	39.8 (37.5, 42.2)	33.6 (31.2, 35.9)	29.9 (27.6, 32.3)	<0.001	0.27 (0.19, 0.39)	<0.001
FLI >60 [n (%)]	132 (34.5)	87 (22.7)	60 (15.6)	60 (15.7)			

Total n-3 PUFA (%)	<4.3	4.3-5.0	5.1-6.0	>6.0			
Model 1	41.3 (38.8, 43.8)	35.0 (32.5, 37.5)	37.1 (34.6, 39.6)	37.7 (35.2, 40.2)	0.241	0.85 (0.62, 1.18)	0.778
Model 2	42.5 (40.1, 45.0)	36.3 (33.8, 38.7)	36.8 (34.3, 39.2)	35.6 (33.1, 38.0)	0.001	0.67 (0.48, 0.95)	0.081
FLI >60 [n (%)]	105 (27.4)	67 (17.5)	74 (19.3)	93 (24.3)			
ALA (%)	<0.58	0.58-0.72	0.73-0.88	>0.88			
Model 1	40.7 (38.1, 43.2)	35.4 (32.9, 37.9)	35.8 (33.3, 38.3)	39.2 (36.6, 41.7)	0.649	0.94 (0.84, 1.05)	0.398
Model 2	40.2 (37.7, 42.7)	35.9 (33.4, 38.3)	35.6 (33.2, 38.1)	39.4 (36.9, 41.9)	0.883	0.96 (0.85, 1.07)	0.556
FLI >60 [n (%)]	103 (26.9)	70 (18.3)	76 (19.8)	90 (23.5)			
EPA (%)	<1.1	1.1-1.4	1.5-1.9	>1.9			
Model 1	40.7 (38.2, 43.2)	36.7 (34.2, 39.2)	35.7 (33.2, 38.2)	38.0 (35.5, 40.5)	0.285	0.91 (0.65, 1.27)	0.873
Model 2	41.5 (39.1, 44.0)	37.5 (35.0, 39.9)	35.6 (33.2, 38.1)	36.5 (34.0, 38.9)	0.011	0.77 (0.55, 1.09)	0.235
FLI >60 [n (%)]	97 (25.3)	77 (20.1)	75 (19.5)	90 (23.5)			
DPA (%)	<0.49	0.49-0.55	0.56-0.63	>0.63			
Model 1	47.7 (45.2, 50.1)	37.6 (35.2, 40.0)	34.2 (31.7, 36.6)	31.7 (29.3, 34.1)	<0.001	0.34 (0.24, 0.48)	<0.001
Model 2	47.1 (44.7, 49.5)	37.9 (35.5, 40.2)	34.3 (31.9, 36.7)	31.8 (29.4, 34.2)	<0.001	0.35 (0.24, 0.50)	<0.001
FLI >60 [n (%)]	130 (33.9)	82 (21.4)	70 (18.2)	57 (14.9)			
DHA (%)	>2.0	2.0-2.3	2.4-2.9	>2.9			
Model 1	38.8 (36.3, 41.3)	36.6 (34.1, 39.2)	38.4 (35.9, 40.9)	37.2 (34.7, 39.7)	0.572	0.96 (0.69, 1.34)	0.972
Model 2	41.1 (38.6, 43.6)	37.2 (34.8, 39.7)	38.1 (35.7, 40.5)	34.6 (32.1, 37.1)	0.001	0.67 (0.47, 0.97)	0.053
FLI >60 [n (%)]	92 (24.0)	78 (20.4)	80 (20.8)	89 (23.2)			
D6D activity	<0.008	0.008-0.010	0.011-0.014	>0.014			
Model 1	30.2 (27.7, 32.6)	34.6 (32.1, 37.0)	39.4 (36.9, 41.8)	47.0 (44.6, 49.5)	<0.001	3.31 (2.31, 4.76)	<0.001
Model 2	29.8 (27.4, 32.1)	34.2 (31.8, 36.5)	39.4 (37.1, 41.8)	47.7 (45.4, 50.1)	<0.001	3.86 (2.65, 5.62)	<0.001
FLI >60 [n (%)]	52 (13.5)	75 (19.6)	82 (21.4)	130 (33.9)			
D5D activity	<2.96	2.96-3.47	3.48-4.17	>4.17			

Model 1	46.0 (43.6, 48.5)	40.7 (38.3, 43.1)	35.6 (33.1, 38.0)	28.8 (26.4, 31.3)	<0.001	0.30 (0.21, 0.44)	<0.001
Model 2	49.1 (46.8, 51.4)	42.0 (39.7, 44.3)	34.7 (32.4, 37.0)	25.3 (22.9, 27.6)	<0.001	0.17 (0.11, 0.25)	<0.001
FLI >60 [n (%)]	122 (31.9)	97 (25.3)	72 (18.8)	48 (12.5)			

[1]Values are means (95% confidence interval) from the analysis of covariance.

[2]Values are odds ratios (95% confidence interval) from the logistic regression.

Model 1 adjusted for age and examination year.

Model 2 adjusted for Model 1 and leisure-time physical activity (kcal/d), smoking (never smoker, previous smoker, current smoker <20 cigarettes/day and current smoker ≥20 cigarettes/day), and intakes of alcohol (g/wk), energy (kcal/d), carbohydrates (E%) and saturated fatty acids (E%).

Abbreviations: AA, arachidonic acid (C20:4n-6); ALA, alpha-linolenic acid (C18:3n-3); D5D, delta-5-desaturase; D6D delta-6-desaturase; DGLA, dihomo-gamma-linolenic acid (C20:3n-6); DHA, docosahexaenoic acid (C22:6n-3); DPA, docosapentaenoic acid (C22:5n-3); E% percent of energy; EPA, eicosapentaenoic acid (C20:5n-3); FLI, fatty liver index; GLA, gamma-linolenic acid (C18:3n-6); LA, linoleic acid (C18:2n-6); PUFA, polyunsaturated fatty acids

Supplemental table 4. Mean values of fatty liver index and odds for hepatic steatosis in quartiles of serum n-3 and n-6 polyunsaturated fatty acids and delta-6-desaturase and delta-5-desaturase activities in cross-sectional analyses with data from the examinations in 1998-2001

	Quartile of serum polyunsaturated fatty acids						
	1 (n=386)	2 (n=386)	3 (n=386)	4 (n=386)	*P* for trend	Odds ratio (95% CI) for hepatic steatosis (FLI >60) in the highest vs. lowest serum PUFA quartile	*P* for trend
Total n-6 PUFA (%)	<29.5	29.5-32.2	32.3-34.4	>34.4			
Model 1	62.8 (60.5, 65.1)[1]	46.0 (43.7, 48.4)	35.6 (33.3, 37.9)	23.4 (21.0, 25.7)	<0.001	0.04 (0.02, 0.06)[2]	<0.001
Model 2	62.9 (60.6, 65.2)	45.7 (43.4, 48.0)	35.8 (33.6, 38.1)	23.4 (21.1, 25.7)	<0.001	0.03 (0.02, 0.06)	<0.001
FLI >60 [n (%)]	239 (61.9)	120 (31.1)	61 (15.8)	23 (6.0)			
LA (%)	<22.1	22.1-24.5	24.6-26.9	>26.9			
Model 1	61.0 (58.6, 63.3)	46.7 (44.3, 49.1)	36.2 (33.9, 38.6)	23.9 (21.5, 26.3)	<0.001	0.04 (0.03, 0.07)	<0.001
Model 2	60.8 (58.5, 63.2)	46.4 (44.1, 48.8)	36.5 (34.1, 38.8)	24.1 (21.7, 26.5)	<0.001	0.04 (0.03, 0.07)	<0.001
FLI >60 [n (%)]	225 (58.3)	121 (31.3)	71 (18.4)	26 (6.7)			
GLA (%)	<0.24	0.24-0.32	0.33-0.41	>0.41			
Model 1	33.7 (31.0, 36.4)	42.5 (39.8, 45.1)	44.0 (41.3, 46.6)	47.7 (45.0, 50.4)	<0.001	2.22 (1.59, 3.11)	<0.001
Model 2	33.8 (31.1, 36.5)	42.1 (39.4, 44.7)	44.2 (41.5, 46.8)	47.8 (45.1, 50.4)	<0.001	2.25 (1.60, 3.17)	<0.001
FLI >60 [n (%)]	73 (18.9)	113 (29.3)	131 (33.9)	126 (32.6)			
DGLA (%)	<1.16	1.16-1.33	1.34-1.53	>1.53			
Model 1	35.1 (32.4, 37.8)	40.8 (38.1, 43.5)	43.7 (41.1, 46.4)	48.1 (45.4, 50.8)	<0.001	2.04 (1.47, 2.81)	<0.001
Model 2	34.3 (31.6, 36.9)	40.5 (37.9, 43.2)	44.0 (41.3, 46.6)	49.1 (46.4, 51.7)	<0.001	2.27 (1.63, 3.17)	<0.001
FLI >60 [n (%)]	88 (22.8)	111 (28.8)	109 (28.2)	135 (35.0)			
AA (%)	<5.0	5.0-5.7	5.8-6.5	>6.5			
Model 1	49.4 (46.7, 52.1)	43.1 (40.4, 45.8)	38.9 (36.2, 41.6)	36.4 (33.7, 39.1)	<0.001	0.39 (0.29, 0.55)	<0.001
Model 2	50.6 (47.9, 53.3)	43.4 (40.8, 46.0)	39.1 (36.4, 41.7)	34.7 (32.1, 37.4)	<0.001	0.31 (0.22, 0.44)	<0.001
FLI >60 [n (%)]	155 (40.2)	115 (29.8)	94 (24.4)	79 (20.5)			

Total n-3 PUFA (%)	<4.8	4.8-5.7	5.8-6.8	>6.8			
Model 1	44.9 (42.2, 47.6)	43.9 (41.2, 46.6)	42.6 (39.9, 45.3)	36.4 (33.7, 39.1)	<0.001	0.55 (0.40, 0.77)	<0.001
Model 2	46.3 (43.5, 49.0)	44.3 (41.7, 47.0)	42.0 (39.3, 44.6)	35.2 (32.5, 38.0)	<0.001	0.50 (0.35, 0.70)	<0.001
FLI >60 [n (%)]	131 (33.9)	116 (30.1)	113 (29.3)	83 (21.5)			
ALA (%)	<0.78	0.78-0.93	0.94-1.13	>1.13			
Model 1	41.6 (38.8, 44.3)	42.2	40.8	43.3 (40.5, 46.0)	0.456	0.92 (0.67, 1.25)	0.499
Model 2	41.0 (38.3, 43.8)	41.8	41.4	43.6 (40.9, 46.3)	0.209	0.99 (0.72, 1.37)	0.953
FLI >60 [n (%)]	117 (30.3)	112 (29.0)	100 (25.9)	114 (29.5)			
EPA (%)	<1.0	1.0-1.3	1.4-1.9	>1.9			
Model 1	43.0 (40.3, 45.8)	42.8 (40.1, 45.5)	43.4 (40.6, 46.1)	38.6 (35.9, 41.3)	0.016	0.69 (0.50, 0.95)	0.046
Model 2	44.3 (41.6, 47.0)	43.1 (40.4, 45.8)	42.8 (40.1, 45.5)	37.6 (34.9, 40.4)	<0.001	0.62 (0.44, 0.86)	0.010
FLI >60 [n (%)]	126 (32.6)	104 (26.9)	120 (31.1)	93 (24.1)			
DPA (%)	<0.65	0.65-0.75	0.76-0.85	>0.85			
Model 1	48.1 (45.4, 50.9)	42.6 (39.9, 45.3)	39.4 (36.7, 42.1)	37.6 (34.9, 40.4)	<0.001	0.52 (0.37, 0.71)	<0.001
Model 2	48.3 (45.6, 51.1)	42.5 (39.8, 45.2)	39.5 (36.9, 42.2)	37.4 (34.7, 40.2)	<0.001	0.52 (0.37, 0.72)	<0.001
FLI >60 [n (%)]	146 (37.8)	111 (28.8)	92 (23.8)	94 (24.4)			
DHA (%)	<2.1	2.1-2.5	2.6-3.2	>3.2			
Model 1	46.9 (44.2, 49.6)	44.0 (41.3, 46.7)	39.7 (37.0, 42.4)	37.3 (34.6, 40.0)	<0.001	0.51 (0.37, 0.70)	<0.001
Model 2	48.2 (45.5, 51.0)	44.4 (41.7, 47.0)	39.3 (36.6, 41.9)	35.9 (33.2, 38.7)	<0.001	0.44 (0.31, 0.62)	<0.001
FLI >60 [n (%)]	141 (36.5)	113 (29.3)	104 (26.9)	85 (22.0)			
D6D activity	<0.010	0.010-0.013	0.014-0.018	>0.018			
Model 1	30.7 (28.1, 33.3)	38.4 (35.8, 40.9)	45.1 (42.5, 47.6)	53.7 (51.1, 56.3)	<0.001	5.02 (3.52, 7.15)	<0.001

Model 2	31.0 (28.4, 33.6)	38.2 (35.6, 40.7)	45.0 (42.4, 47.5)	53.7 (51.1, 56.3)	<0.001	5.01 (3.50, 7.17)	<0.001
FLI >60 [n (%)]	55 (14.2)	92 (23.8)	128 (33.2)	168 (43.5)			
D5D activity	<3.5	3.5-4.2	4.3-5.2	>5.2			
Model 1	53.1 (50.5, 55.7)	45.0 (42.4, 47.6)	37.9 (35.3, 40.5)	31.8 (29.2, 34.4)	<0.001	0.23 (0.17, 0.33)	<0.001
Model 2	55.2 (52.6, 57.8)	45.0 (42.4, 47.5)	37.9 (35.4, 40.5)	29.7 (27.2, 32.3)	<0.001	0.17 (0.11, 0.24)	<0.001
FLI >60 [n (%)]	169 (43.8)	127 (32.9)	85 (22.0)	62 (16.1)			

[1]Values are means (95% confidence interval) from the analysis of covariance.
[2]Values are odds ratios (95% confidence interval) from the logistic regression.
Model 1 adjusted for age, gender and examination year.
Model 2 adjusted for Model 1 and leisure-time physical activity (kcal/d), smoking (never smoker, previous smoker, current smoker <20 cigarettes/day and current smoker ≥20 cigarettes/day), and intakes of alcohol (g/wk), energy (kcal/d), carbohydrates (E%) and saturated fatty acids (E%).
Abbreviations: AA, arachidonic acid (C20:4n-6); ALA, alpha-linolenic acid (C18:3n-3); D5D, delta-5-desaturase; D6D delta-6-desaturase; DGLA, dihomo-gamma-linolenic acid (C20:3n-6); DHA, docosahexaenoic acid (C22:6n-3); DPA, docosapentaenoic acid (C22:5n-3); E%, percent of energy; EPA, eicosapentaenoic acid (C20:5n-3); FLI, fatty liver index; GLA, gamma-linolenic acid (C18:3n-6); LA, linoleic acid (C18:2n-6); PUFA, polyunsaturated fatty acids
